# How to Keep Well in Africa

**Published:** 1893-05-27

**Authors:** 


### HOW TO KEEP WELL IN AFRICA.

### II.—CURE.

WE have already shown that to keep well in Africa one's motto must be this : "Be bold, be bold, be not too bold." As a pendant to that we might add: "Take quinine, take quinine, don't take too much quinine." In Africa everyone needs quinine now and then; but it does not follow that it is a good principle to be continually "nipping" at quinine any more than at whisky, which also has its place in African life. No drug, however valuable in its place, was meant to be "human nature's daily food."

Nevertheless, as in speaking of African fever one must again and again speak of quinine, it is difficult to make it clear within what limits it should be used. In Africa everyone has fever, and the name soon ceases to be a word of fear. When one has met men who have, like one known to Mr. Waller, undergone eighty attacks of fever in three years, and survived them, one begins to think that fever means something very different in the tropics from what it does in the temperate zone. And it is true that in some cases fever means no more serious ailment than would a common cold with us, yet it is not on that account safe to regard African fever as a trifling disease. African fever may be divided into three classes: intermittent, remittent, and hæmaturic fever. The first of these is the commonest and least dangerous, though not on that account to be trifled with. Fever is always on the alert; so should man be. Where malaria is fever will ever be. And malaria is nearly everywhere. It lurks in the sand of dry water-courses, which in wet seasons are river-beds. It creeps up through cracks in the soil, the outcome of decaying vegetation far below. It floats over stagnant water, and lingers about the ground, though sometimes its evil effects do not develop until the purer air of hill or plateau drives it out in an attack of fever, which, however, will prove of a milder character than it would otherwise have been. But, indeed, the poison is always there, and a chill whilst sleeping, wet feet, even an indigestible meal, or a restless night, will serve to give it the excuse which it desires.

The inexperienced victim does not know when he is first attacked. He feels well, cheerful, excitably exuberant. True, these symptoms are ready to change on the smallest provocation to irritability, and soon pass into fretful lassitude. Then he becomes sick and cold, and no matter how hot the day, no matter how many blankets and wraps are piled on him, he shivers as if he were at the pole instead of the Equator. This lasts for an hour or more, and then comes the hot stage, in which, like Madame Roland, one would fain write down the strange thoughts that arise in one's mind, but cannot attain the philosophic calm. Memory and imagination are brilliantly active, but withal the patient is acutely miserable until the stage of perspiration comes, when the poison that has been throbbing in his veins escapes through the pores of the skin. Sometimes the very scent of the mangrove swamps, where the poison was imbibed, may be detected in that perspiration.

The paroxysm is over, but the fever is not past. Within twenty four hours (in quotidian ague) or forty-eight (in tertian ague) the same symptoms will return, and if left unchecked will go on indefinitely, leaving the patient weaker every time, even though between the attacks he is able to get up and work. There fore, as soon as the first sickness has subsided administer pills made as follows: Calomel, eight grains; resin of jalap, eight grains; pulverised rhubarb, six grains; quinine, six grains. Make into pills with a few drops of tincture of cardamons. It is not desirable to carry the pills ready-made, unless, indeed, they can be had coated, as they are liable to become mouldy. The dose is from six to twelve grains of the pill mass, and must be regulated by the patient's constitution. If he is susceptible to aperients the smaller dose will do; according to any tendency to constipation it must be increased. The intention of the medicine is to secure elimination through the bowels; when this has been accomplished, which should be within five hours, a dose of five grains of quinine should be administered every four hours for the next twelve hours. The worst cases are those in which the bowels are inactive. If the pills fail of result resort must be had to senna tea, salts, or castor oil. If these fail it is evidently useless to administer more medicine; the cause of inaction is a sort of paralysis of the intestine, and more aperients may cause distress without doing any good. Now is the time when an enema is of use. Three ounces of castor oil to two pints of warm soapy water will work wonders where all drugs have failed. An enema apparatus should always be included in the baggage, and in an attainable part of it; and the simpler its kind, the less liable to get out of order, the better.

Since constipation is a pronounced evil in fever cases, its opposite is, though not obviously, a good. Diarrhœa is nature's attempt to eliminate the fever-poison, and unless it developes into dysentery, it had better not be checked. There is always considerable pain in the back when this becomes severe; a mild mustard poultice may be applied, but not so strong a mixture as would be used in England. Cool effervescing drinks help to relieve the distress of the hot stage, and cool and hot drinks administered internally tend to promote perspiration. For the rest, the patient may drink coffee or weak tea, but, unless he is threatened with collapse, he is better without wine or spirits. It is a great matter, and one in which the need of careful watching comes in, to decide when stimulants are necessary. In so far as they promote congestion of the liver they are bad, but in cases of extreme weakness this may be the less of two evils. At a certain point death may result from anæmia of the brain. A weak patient makes some trifling effort, perhaps only rises from his chair to walk a few yards, but the exertion withdraws the blood from the brain, and he faints. In such a case hold his head below the level of the body to restore the blood, and at once administer some wine—champagne by preference, if it is attainable. In such cases wine, ale, and brandy, should always be at hand. They are necessary medicines, not luxuries, not even comforts.

The worst form of fever is haematuric fever. This is, pathologically, the kidneys taking upon themselves the task of eliminating the miasma poison, and proving themselves unequal to it. In this form there is incessant sickness, accompanied by jaundice. There is hæmorrhage from the kidneys, and a destruction of blood-tissue throughout the body, the malignant action of the malaria poison. When these symptoms appear, steel drops should be administered, and a diuretic may be serviceable. Hypodermic injection of ergot has been found useful, but this should be administered only to men. The hypodermic syringe is a dangerous friend. Quinine has been injected in this way with good effect, but it is well to know that sometimes tetanus has resulted from it, when a nerve has been injured by the needle, or the injection has entered a vein.

The principles to be adhered to in dealing with African fever are—(1) relieve the bowels, (2) induce perspiration, (3) keep up the patient's strength. The pills for which we have given the prescription are as reliable as anything for the first purpose. They were strongly recommended by Dr. Livingstone, and several who have at first taken up other preparations have finally given in their adhesion to these. For the second a mixture in which General Gordon placed implicit faith, called Tinctura Warburgi, or "Warburg's Drops," is said to be "the most powerful sudorific known." For the third, quinine. Antipyrine has been by many too thoughtlessly recommended. It, indeed, relieves headache and reduces the temperature, but its depressing after effects condemn it, and it is apt to affect the heart. If, in spite of the use of quinine, there is any threatening of coma, stimulants must be used, and it has been found that bitter ale is sometimes retained when all else is rejected. But unless there are symptoms of collapse, it is better to avoid spirits, and rely on tea, coffee, broth, and effervescing drinks to relieve the patient's thirst.

